# Editorial: Myeloid cell reprogramming: molecular pathways involved in disease development

**DOI:** 10.3389/fimmu.2024.1414482

**Published:** 2024-04-30

**Authors:** Denise Risnik, Ana Colado, Enrique Podaza

**Affiliations:** ^1^ Instituto de Farmacología, Facultad de Medicina, Universidad de Buenos Aires, Buenos Aires, Argentina; ^2^ Laboratorio de Inmunología Oncológica, Instituto de Medicina Experimental (IMEX)-Consejo Nacional de Investigaciones Científicas y Técnicas (CONICET)- Academia Nacional de Medicina (ANM), Buenos Aires, Argentina; ^3^ Caryl and Israel Englander Institute for Precision Medicine, Weill Cornell Medicine, New York, NY, United States

**Keywords:** myeloid cells, trained immunity, macrophages, myeloid cell reprogramming, MDSC (myeloid-derived suppressor cell)

Since they were first described, our knowledge of myeloid cells has grown considerably, allowing us to further categorize the different types of these cells such as monocytes, macrophages, dendritic cells, neutrophils and myeloid-derived suppressor cells (MDSC). It is now known that each of these cells also have different subtypes and profiles, highly influenced by the physiological or pathophysiological environment in which they are immersed. This plasticity is a key characteristic of myeloid cells and a determining factor of their function.

The environment’s modulation over myeloid cell subsets was initially thought of as a transitional state. Nevertheless, it is presently established that it can also impart a persistent influence on the myeloid compartment. This phenomenon is known as trained immunity, a different type of immune memory than the one developed by adaptative immunity (1). In this sense, Kweon et al. illustrate in their review how maternal obesity can imprint on the unborn’s myeloid cells, generating a broad spectrum of clinical future conditions even in adult life. These conditions range from metabolic disorders to psychiatric alterations, inflammatory disease, and susceptibility to different pathogens among others.

The better we understand the impact of different stimuli on the subsets and behavior of myeloid cells, the closer we can come to manipulating them for therapeutic purposes. Ortiz Wilczyñski et al. focused on the problem of chronic wounds where the resolution of the inflammation and healing is hampered. They demonstrated through a series of experiments that the synthetic ceramide C8-C1P, but not the natural (C16-C1P), can improve chronic wound healing by priming the monocyte compartment, leading to macrophages with longer survival and higher angiogenic, anti-inflammatory, and tissue repair properties.

One area of research where immunotherapy has had a larger impact is cancer research. The development of immune checkpoint inhibitors has become a milestone in cancer treatment. These monoclonal antibodies allow the restitution of antitumor T cell responses that were inhibited by malignant cells and the tumor microenvironment (2). Although this particular strategy focuses primarily on T cells, we understand the immune system as an intricate network where leukocyte interactions play a key role. Myeloid cells are also critical targets in cancer therapy. Therefore, Alcantara et al. hypothesize and demonstrate that MDSC reprogramming may not only have direct therapeutic benefits but also significantly improve the outcomes of immune checkpoint blockade in non-responsive patients. As shown in this original article, targeted inhibition of STAT3 in myeloid cells by CpG-STAT3ASO can not only reduce the immunosuppressive activity of MDSC but also activate dendritic cells, favor the M1 macrophage profile over M2 and indirectly improve the CD8 to regulatory T cell ratio.

Furthermore, another cancer treatment that involves the immune system as part of its mechanism of action is ionizing radiation (IR), as it induces immunogenic cell death of irradiated tumor cells (3). After radiation, the damage signals that are released attract myeloid cells to the tissue, such as dendritic cells, and monocytes that then differentiate into macrophages. Mikhalkevich et al. aim to deepen the understanding of the mechanism involved in the programming of macrophages after IR. As well demonstrated here, the nucleotide species generated after low or high doses of IR are not the same and, consequently, neither are the resulting macrophages and their pro- or anti-inflammatory properties.

While Myeloid cell reprogramming, particularly focusing on MDSCs and macrophages, has emerged as a focal point in cancer research, its significance extends beyond oncology, as these cells play a fundamental role in various pathological processes, including infections. In this regard, Zhang et al. have reviewed the advances on this subject in pulmonary infectious diseases with special focus on COVID-19 pneumonia and tuberculosis where the amount of MDSC correlates with greater inflammation and worse outcomes. MDSCs are known for their immune suppressive functions, notwithstanding, Xie et al. also demonstrated on murine models of renal ischemia-reperfusion injury and aging, that these cells are responsible for higher inflammatory responses to viral pneumonia, highlighting once more how the context is a key determinant of myeloid cell activity.

Moreover, Zec et al. used a transcriptomic and proteomic approach to study the adaptation of alveolar macrophages to a common pulmonary bacterial infection, such as Streptococcus pneumoniae pneumonia. The results show a differential expression of several molecules in alveolar macrophages of mice after infection, where CD11b stands out as a central protein in this adaptation process, modulating macrophage activation, antigen presentation, phagocytosis and even the recruitment of neutrophils, another important myeloid cell in bacterial infections.

Collectively, this Research Topic has brought forth diverse strategies to better understand and harness myeloid cell plasticity and trained immunity with potential translational benefits.

## Author contributions

DR: Writing – original draft, Writing – review & editing. AC: Writing – original draft, Writing – review & editing. EP: Writing – original draft, Writing – review & editing.
